# *In vitro* and *in vivo* efficacy of aurintricarboxylic acid against *Neospora caninum* infection: Efficacy of aurintricarboxylic acid against *N*. *caninum* infection

**DOI:** 10.3724/abbs.2025006

**Published:** 2025-03-28

**Authors:** Zhengkai Wei, Yuxiao Qian, Xi Jiang, Yuqian Jiang, Rongsheng Huang, Kaifeng He, Jing Huang, Jiaxuan Wang, Xin Guo, Wenlong Huang, Dezhi Zhang, Zhengtao Yang, Quan Liu, Qianyong Li

**Affiliations:** 1 College of Veterinary Medicine Southwest University Chongqing 400715 China; 2 School of Animal Science and Technology Foshan University Foshan 528225 China

**Keywords:** *Neospora caninum*, aurintricarboxylic acid, bovine, macrophage

## Abstract

Bovine neosporosis, a protozoal disease caused by *Neospora caninum* ( *N*. *caninum*), poses a significant threat to the global cattle industry, resulting in substantial economic losses that are difficult to quantify. The current lack of effective commercial vaccines and specific treatments highlights the urgent need for the development of potent drugs against *N*. *caninum*. In this study, we investigate the efficacy of aurintricarboxylic acid (ATA), a derivative of polyaromatic carboxylic acid, against *N*. *caninum* both *in vitro* and *in vivo*. Cell cytotoxicity is evaluated using CCK-8 kits. *N*. *caninum* proliferation within cells is assessed by qPCR analysis. Transmission electron microscopy (TEM) is employed to examine the ultrastructures of *N*. *caninum* tachyzoites. The efficacy of ATA against *N*. *caninum* infection is validated in a mouse model. Our findings indicate that ATA not only inhibits *N*. *caninum* proliferation but also reduces parasite loads within individual cells. Furthermore, ATA (20 and 40 μM) has immunomodulatory effects by downregulating the mRNA expressions of *N*. *caninum*-induced cytokines, including tumor necrosis factor-α (TNF-α), interferon (IFN-α, -β, and -γ), and β-defensin 5 (BNBD5). ATA treatment directly targets and eliminates *N*. *caninum* by disrupting its ultrastructure. The *in vivo* study confirms the potential of ATA to increase body weight, decrease parasite loads in the lungs and duodenum, and ameliorate the pathological effects induced by *N*. *caninum* infection in mice. In conclusion, this study represents the first evidence of the anti- *N*. *caninum* ability of ATA and provides compelling data to support its potential as a candidate for developing anti- *N*. *caninum* drugs.

## Introduction

*Neospora caninum* ( *N*. *caninum*) is a globally significant apicomplexan parasite that affects cattle and canines, causing reproductive loss in cattle and neuromuscular disease in canines [ 1– 3]. *N*. *caninum* infection in pregnant dairy and beef cows leads to substantial economic losses in global animal husbandry annually [ 3, 4]. Despite the availability of some commercial vaccines with uncertain protective effects on fetuses, there is currently no specific effective treatment for *N*. *caninum*. Therefore, the development of efficacious anti- *N*. *caninum* drugs for neosporosis is imperative.

Cattle serve as intermediate hosts for *N*. *caninum*; however, their large size and high cost make them difficult to use in experiments. Macrophages play an irreplaceable role in fighting *N*. *caninum* infection [ 5, 6], and *N*. *caninum* can parasitize macrophages [7]. Bovine macrophages are valuable cell strains for the study of anti- *N*. *caninum* drugs. Aurintricarboxylic acid (ATA) is a compound that has diverse biological activities, including the inhibition of nucleases [8], the JAK-STAT pathway [9], topoisomerase II and apoptosis [10]. ATA exhibits broad-spectrum antiviral activity against various viruses, including SARS-CoV-2 [11], HIV [ 12, 13], Zika virus [14], and cowpox virus [15]. Moreover, ATA has shown potential efficacy against parasites, including *African trypanosomes* and *Cryptosporidium parvum* [ 16, 17]. However, it remains uncertain whether ATA possesses specific activity against *N*. *caninum-*infected bovine macrophages and a murine model.

In this study, we determined the non-toxic concentration of ATA for bovine macrophages and employed qPCR to demonstrate that ATA significantly reduces *N*. *caninum* loads in bovine macrophages. Subsequently, fluorescence staining revealed that ATA not only inhibited the proliferation of *N*. *caninum* but also decreased the parasite load within individual cells. We evaluated cytokine levels in bovine macrophages and found that ATA significantly decreases the mRNA levels associated with the *N*. *caninum*-induced upregulation of cytokines. Moreover, fluorescence staining combined with TEM confirmed the direct killing effect of ATA on *N*. *canium*. Finally, the efficacy of ATA against *N*. *caninum* infection was validated in a murine model. In conclusion, this study elucidates the potent anti- *N*. *caninum* activity of ATA, providing a foundation for the future development of anti- *N*. *caninum* therapeutics.

## Materials and Methods

### Culture and purification of *N*. *caninum* tachyzoites

Bovine macrophages were maintained in our laboratory as previously described [ 5, 18]. *N*. *caninum* tachyzoites (strain Nc-1) were inoculated into Vero cells at 80% density and incubated with RPMI Medium 1640 (Solarbio, Shanghai, China) containing 2% fetal bovine serum (FBS). Following extrusion of the majority of *N*. *caninum* parasites from the host cells, purification was carried out via a previously described method [ 19– 22]. Infected VERO cells were centrifuged at 1580 *g* for 10 min to harvest *N*. *caninum* tachyzoites. The pellet was collected and resuspended in RPMI 1640 medium and then sequentially passed through a 5-mL syringe, a1-mL syringe, and a 27-gauge needle. Finally, *N*. *caninum* tachyzoites were purified by centrifugation at 1580 *g* for 30 min on a density gradient consisting of 40 % Percoll (GE, Shanghai, China). After two washes, the resulting pellet was resuspended in RPMI 1640 medium.

### Cell counting kit-8 (CCK-8) assay

Bovine macrophages were seeded into 96-well plates at a density of 5 × 10 ^4^ per well and incubated with different concentrations (0, 5, 10, 20, 40, or 80 μM) of ATA (MCE, Monmouth Junction, USA) in RPMI Medium 1640 for 24 h at 37°C with 5% CO _2_. Cell viability was assessed using Cell Counting Kit-8 (GLPBIO, Montclair, USA) following the manufacturer’s instructions.

### Detection of *N*. *caninum* quantity in bovine macrophages

Bovine macrophages were inoculated into a 6-well plate at a density of 1 × 10 ^6^ per well and pre-treated with different concentrations of ATA (20, 40, or 80 μM) or RPMI Medium 1640 for 1 h. Subsequently, the cells were infected with *N*. *caninum* at a cell-to-parasite ratio of 1:1. After co-cultivation for 24 h at 37°C with 5% CO _2_, the cells were gently washed three times with PBS to remove the extracellular *N*. *caninum*. After cell morphology examination and collection of images, the cells were harvested using cell scrapers and transferred to 1.5-mL microtubes for DNA extraction.

DNA extraction was performed via a TIANamp Genomic DNA kit (TIANGEN, Beijing, China) following the manufacturer’s instructions. Total DNA (200 ng) from each sample was used as a template for the qPCR analysis. A primer pair specific for the Nc5 sequence of *N*. *caninum* (forward: 5′-ACTGGAGGCACGCTGAACAC-3′, reverse: 5′-AACAATGCTTCGCAAGAGGAA-3′) was used to amplify a 76-bp DNA fragment [23].

### Examination of *N*. *caninum* in bovine macrophages

Bovine macrophages were treated with ATA (40 μM) for 1 h, followed by infection with *N*. *caninum* tachyzoites of the Nc-1 GFP strain for 24 h. Subsequently, the cells were stained with the Tubulin-Tracker Deep Red Staining kit for Living Cells (Beyotime, Shanghai, China) and Hoechst 33342 (Sigma-Aldrich, St Louis, USA). A confocal laser scanning microscope (CLSM; ZEISS, Wetzlar, Germany) was used to observe the samples. The *N*. *caninum* tachyzoites (Nc-1 GFP) appeared green, while cytoskeleton staining is represented in red, and nuclear staining is represented in blue.

### Analysis of cytokine expression in bovine macrophages

Total RNA was extracted from bovine macrophages using Triquick Reagent (Solarbio) according to the manufacturer’s instructions. The total RNA (2000 ng) obtained from each sample served as a template during reverse transcription via the RevertAid First Strand cDNA Synthesis kit (Thermo Fisher Scientific, Waltham, USA).

qRT-PCR was performed on a Quant Studio 3 Real-Time PCR System (Applied Biosystems, Foster City, USA) using the Roche 25 μL system (cat. 06924204001; Roche, Basel, Switzerland). The reaction conditions consisted of an initial step at 50°C for 2 min, followed by a step at 95°C for 10 min, and then cycling between steps at 95°C for 15 s and at 60°C for 60 s over a total of 40 cycles [24]. Data analysis was performed via the 2 ^–∆∆Ct^ method, with *GAPDH* serving as an internal reference. The primer sequences are listed in Table 1, as previously described [ 25– 27].

**Table:** **Table 1** The sequences of primers used in this study

Name	Sequence (5′→3′)	Product size (bp)
*GAPDH* forward	TCAACGGGAAGCTCACTGG	237
*GAPDH* reverse	CCCCAGCATCGAAGGTAGA	
*BNBD5* forward	GCCAGCATGAGGCTCCATC	143
*BNBD5* reverse	TTGCCAGGGCACGAGATCG	
*TNF-α* forward	CTGCCGGACTACCTGGACTAT	234
*TNF-α* reverse	CCTCACTTCCCTACATCCCTAA	
*IFN-α* forward	GTGAGGAAATACTTCCACAGACTCACT	108
*IFN-α* reverse	TGARGAAGAGAAGGCTCTCATGA	
*IFN-β* forward	CAGCACATCTTCGGCATTCTC	103
*IFN-β* reverse	GACGATTCATCTGCCCATAG	
*IFN-γ* forward	ACCTCCTTGGGACCTGAT	208
*IFN-γ* reverse	CTACATCTGGGCTACTTG	
*IL-6* forward	AACGAGTGGGTAAAGAACGC	144
*IL-6* reverse	CTGACCAGAGGAGGGAATGC	

### Toxicity of ATA to *N*. *caninum*

Live or dead *N*. *caninum* were assessed by exposing them to Hoechst 33342 and SYTOX orange (Thermo Fisher Scientific) and incubating them with varying concentrations of ATA (0, 20, 40, or 80 μM) for different durations ranging from 0 to 240 min. The samples were analyzed using a CLSM. Live and dead *N*. *caninum* stained blue, while dead *N*. *caninum* stained red. Images of five random fields were captured per treatment, with a minimum of 500 *N*. *caninum* observed per field.

### Transmission electron microscopy (TEM)

The ultrastructures of *N*. *caninum* tachyzoites were observed with a transmission electron microscope (HT7700; HITACHI, Tokyo, Japan). In brief, tachyzoites were treated with 1640 or 40 μM ATA for 4 h. Tachyzoites were harvested and centrifuged at 1580 *g* for 10 min. Tachyzoites were fixed in 2.5% glutaraldehyde. After three washes, the samples were postfixed in osmium tetroxide, dehydrated in ethanol, treated with propylene oxide, and embedded in Spurr’s epoxy resin.

### Animals

Female C57BL/6 mice, aged 3–4 weeks, were purchased from the Guangdong Medical Laboratory Animal Center (Foshan, China). The mice were housed in individual isolator cages, ensuring an adequate supply of food and water. Under stringent husbandry conditions, the mice were maintained in standard cages, with a group size of six per cage, under a natural light/dark cycle (12 h light/12 h dark), at a constant temperature of 25°C. The mice were intraperitoneally inoculated with 1 × 10 ^5^ *N*. *caninum* tachyzoites suspended in 100 μL of normal saline solution. The control cohort received an equivalent volume of saline solution. Following infection, the treatment group was administered with oral doses of ATA (25, 50, and 100 μmol/kg) in 100 μL volumes daily for seven consecutive days as previously described [17], beginning on the day of infection. The control group was provided with an equivalent volume of normal saline. Daily measurements of body weight and average food and water intake were recorded. Clinical symptoms were meticulously observed and documented. Upon necropsy, the organs were removed, and their weights were determined. All the experimental procedures were conducted in strict compliance with the standards and practices of the Institutional Animal Care and Use Committee of Foshan University.

### Detection of *N*. *caninum* quantity in organs

Genomic DNA was extracted from the brain, heart, lung, liver, spleen, kidney and duodenum of the mice using a TIANamp Genomic DNA kit (TIANGEN) according to the manufacturer’s instructions. Total DNA (200 ng) from each sample was used as a template for the qPCR analysis. qPCR was performed using the Roche 25 μL system (Roche) under the following cycling conditions: 50°C, 2 min; 95°C, 10 min; 95°C, 15 s; and 60°C, 60 s, for a total of 40 cycles [24]. A primer pair specific for the *Nc5* sequence of *N*. *caninum* was: forward: 5′-ACTGGAGGCACGCTGAACAC-3′ and reverse: 5′-AACAATGCTTCGCAAGAGGAA-3′) [23].

### Aspartate aminotransferase (AST) and alanine aminotransferase (ALT) detection

AST and ALT levels in the liver were quantified using commercially available assay kits from Nanjing Jiancheng Bioengineering Institute (Nanjing, China), and the optical density (OD) was measured with a microplate reader (Infinite 200; TECAN, Männedorf, Switzerland) as previously described [28].

### Blood urea nitrogen (BUN) detection

BUN levels in the serum were assessed using commercial assay kits from Nanjing Jiancheng Bioengineering Institute, and the OD values were determined with a microplate reader as previously described [29].

### Histological examination

Tissue samples from the duodenum, liver, kidney, and lung were collected, fixed in 10% formaldehyde, dehydrated through a graded series of alcohol concentrations, and embedded in paraffin for sectioning. Sections were stained with hematoxylin and eosin, as previously described [ 24, 30]. The histological sections were then examined under a microscope to observe pathological changes.

### Statistical analysis

Data are expressed as the mean ± standard deviation (SD), and GraphPad Prism 9 software was used for data analysis. Statistical differences were evaluated using one-way analysis of variance (ANOVA) with Tukey’s multiple comparison test or two-way analysis of variance with Dunnett’s multiple comparisons test. *N*. *caninum* numbers per cell in the *N*. *caninum* and ATA groups were statistically analyzed via an unpaired Student’s *t* test. *P*  < 0.05 was considered as statistically significant.

## Results

### ATA exhibits minimal cytotoxicity towards bovine macrophages

**Figure 1:**
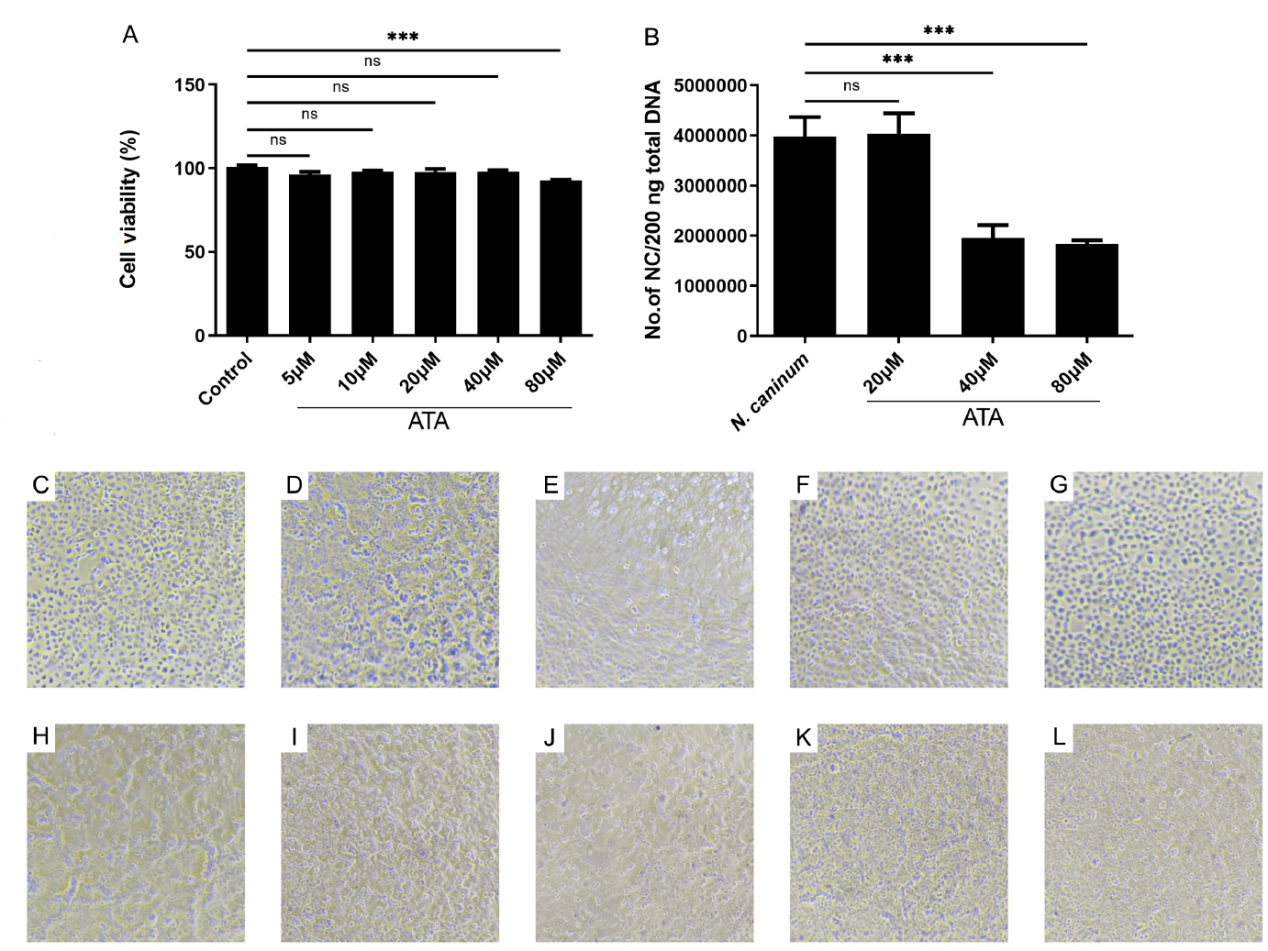
Figure 1 Effects of ATA on bovine macrophage viability and *N*. *caninum* loads within macrophages (A) Effects of ATA on bovine macrophage viability. The cells were cultured with ATA at concentrations of 5, 10, 20, 40 and 80 μM for 24 h. The effects of ATA on cell viability were examined by using Cell Counting Kit-8. There was no significant difference between the control group and ATA at concentrations ranging from 5 to 40 μM; however, a significant decrease in cell viability was observed at an ATA concentration of 80 μM. (B) ATA inhibits the proliferation of N. caninum in bovine macrophages. Bovine macrophages were pretreated with or without ATA at concentrations of 20, 40 and 80 μM, followed by stimulation with N. caninum at a ratio of 1:1 for 24 h. The parasite load was evaluated using qPCR analysis of total DNA extracted from infected macrophages (200 ng). Significantly reduced parasite loads in bovine macrophages were observed with ATA concentrations of 40 and 80 μM. Morphological observation of bovine macrophages. (C,H) Control. (D,I) N. caninum. (E,J) N. caninum + ATA (25 μmol/kg). (F,K) N. caninum + ATA (50 μmol/kg). (G,L) N. caninum + ATA (100 μmol/kg). (C–G) Culture was maintained for 0 h. (H–L) Culture was maintained for 24 h. ***P < 0.001.

Following 24 h of exposure to ATA at various concentrations (5, 10, 20, 40, and 80 μM), only the highest concentration of 80 μM ATA resulted in a modest reduction in cell viability ( Figure 1A). After co-incubation of the cells with *N*. *caninum* and ATA at various concentrations, no significant differences in cell growth were observed ( Figure 1C–L). These findings indicate that ATA has minimal cytotoxic effects on bovine macrophages.

### ATA potently inhibits *N*. *caninum* proliferation in bovine macrophages

To assess the influence of ATA on *N*. *caninum* proliferation within bovine macrophages, cells were pretreated or untreated with ATA at concentrations of 20, 40 and 80 μM prior to a 1:1 ratio co-incubation with *N*. *caninum* for 24 h. qPCR analysis of total DNA extracted from the infected macrophages (200 ng) was performed to evaluate the parasite loads. A significant reduction in parasite load was observed in the presence of ATA at concentrations of 40 and 80 μM ( Figure 1B), suggesting that ATA potently inhibits the proliferation of *N*. *caninum* in bovine macrophages.

### Visual examination of the ability of ATA to inhibit *N*. *caninum* proliferation in bovine macrophages

**Figure 2:**
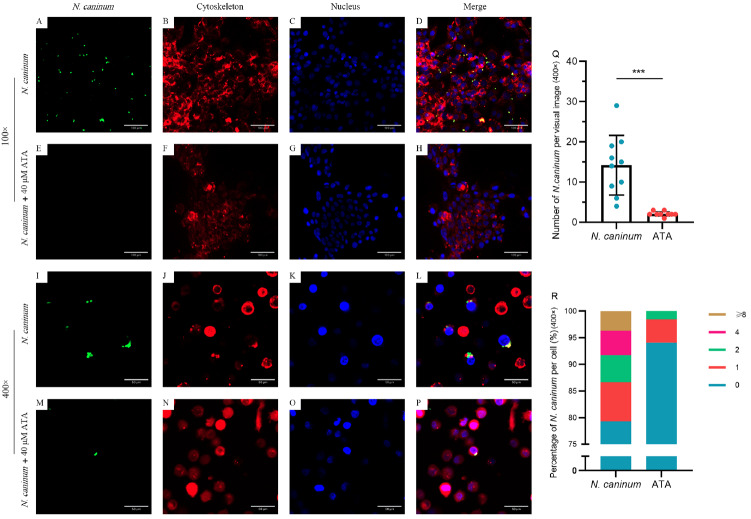
Figure 2 Visual examination of the ability of ATA to inhibit *N*. *caninum* proliferation in bovine macrophages Bovine macrophages were pretreated or untreated with ATA at a concentration of 40 μM, followed by stimulation with N. caninum at a ratio of 1:1 for 24 h. CLSM was employed to visualize the proliferation of N. caninum in bovine macrophages. The N. caninum tachyzoites of Nc-1 GFP appeared green (A, E, I and M), while cytoskeleton staining is represented in red (B, F, J and N), and nuclear staining is represented in blue (C, G, K and O). (Q) Statistical analysis of the total N. caninum number per visual image (400×; n = 9 or 10). (R) Percentages of classified statistically significant N. caninum numbers per cell (400×; n = 9 or 10). This confirmed that ATA possesses anti-N. caninum activity, notably reducing both the overall quantity and individual number per cell of N. caninum. ***P < 0.001.

CLSM was employed to visually examine the proliferation of *N*. *caninum* in bovine macrophages ( Figure 2). The GFP-expressing *N*. *caninum* tachyzoites appeared green ( Figure 2A,E,I,M), while the cytoskeleton and nucleus were stained red ( Figure 2B,F,J,N) and blue ( Figure 2C,G,K,O) respectively. Our observations and statistical analysis confirmed that ATA possesses anti- *N*. *caninum* activity, with notable reductions in both the overall number and the number of parasites per cell ( Figure 2Q,R).

### ATA significantly inhibits *N*. *caninum-*induced mRNA expressions of cytokines and *BNBD5*

**Figure 3:**
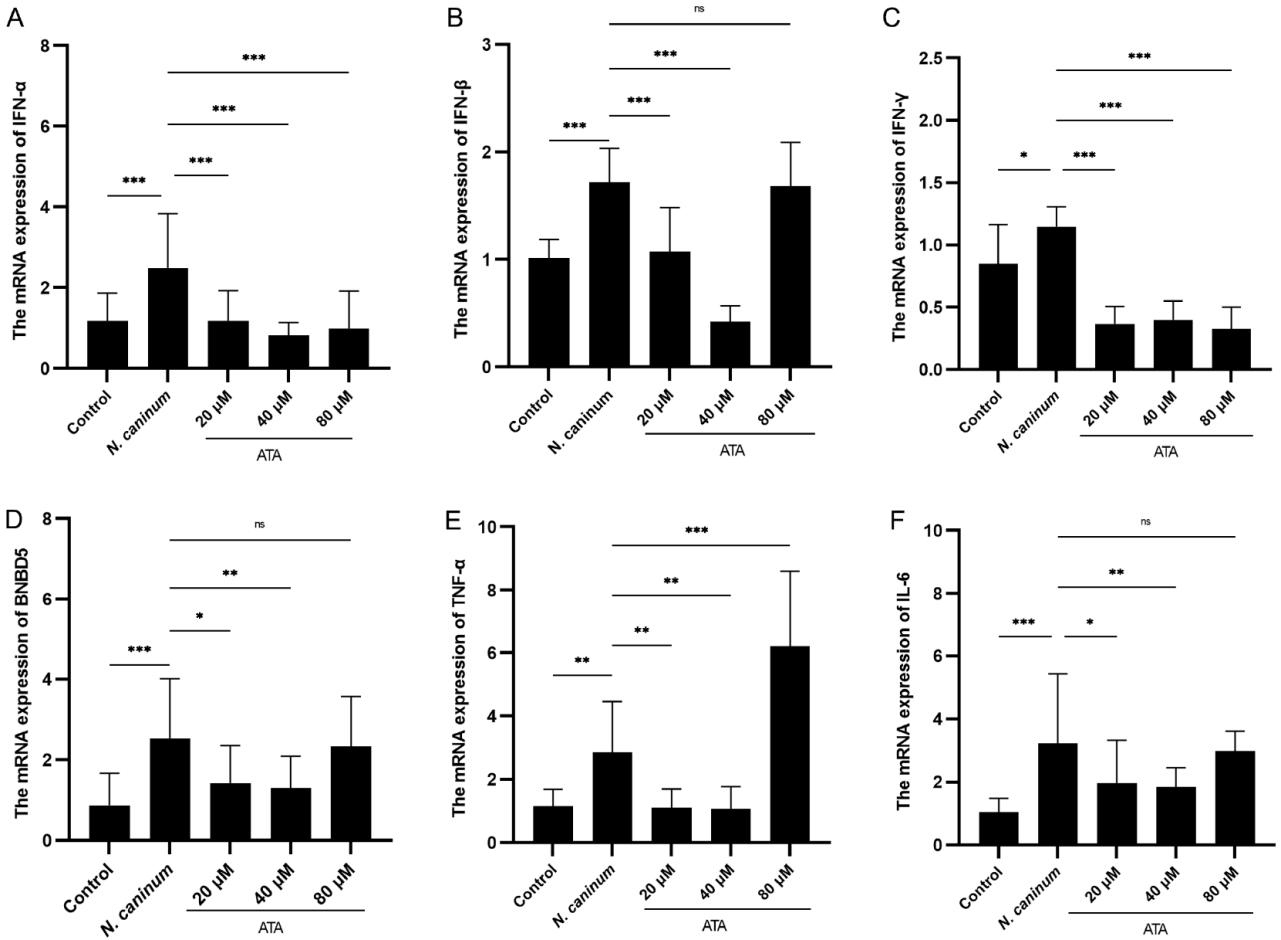
Figure 3 ATA inhibits *N*. *caninum*-induced increases in the mRNA levels of cytokines and *BNBD5* (A) The mRNA expression of IFN-α. (B) The mRNA expression of IFN-β. (C) The mRNA expression of IFN-γ. (D) The mRNA expression of BNBD5. (E) The mRNA expression of TNF-α. (F) The mRNA expression of IL-6. The differences among groups were analyzed by one-way analysis of variance (ANOVA) and Tukey’s multiple comparison test. Data are presented as the mean ± SD (n = 5). *P < 0.05, **P < 0.01, ***P < 0.001, and ns indicates no statistical significance.

Given the pivotal role of bovine macrophages in immune response regulation, the effect of ATA on cytokine expression profiles altered by *N*. *caninum* was examined by qRT-PCR analysis. Our findings revealed significant upregulation of *TNF*- *α*, *BNBD5*, *IL*- *6*, and *IFN* (- *α*, - *β*, and - *γ*) expressions upon infection with *N*. *caninum*. However, ATA treatment markedly reduced these expression levels ( Figure 3).

### ATA effectively eliminates *N*. *caninum*

**Figure 4:**
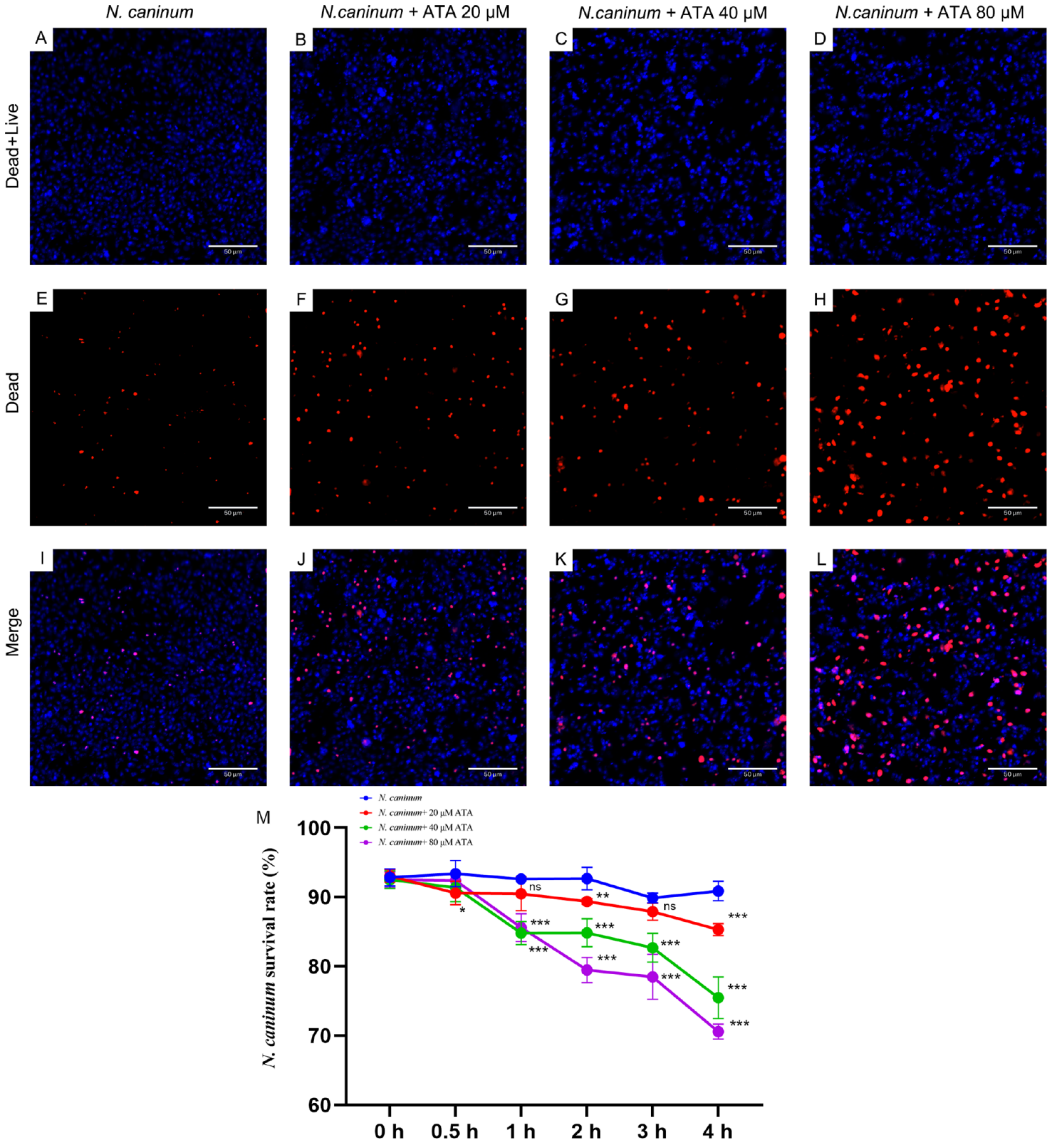
Figure 4 Effects of ATA on the survival status of *N*. *caninum* (A–L) ATA effectively eradicates N. caninum. The effects of ATA on the survival status of N. caninum were evaluated by using Hoechst 33342 and SYTOX orange dyes. Hoechst 33342 (blue) stains both live and dead N. caninum, whereas SYTOX orange (orange) stains only dead N. caninum. As the concentration of ATA increased, the number of dead N. caninum increased. (M) Statistical analysis of the effect of ATA on the survival status of N. caninum. The effects of ATA on the survival status of N. caninum were evaluated within a 4-h timeframe using Hoechst 33342 and SYTOX orange dyes. Statistical analysis revealed that the killing effects of ATA on N. caninum were dependent on both time and dosage within a 4 h period. Data are presented as the mean ± SD (n = 5). **P < 0.01, ***P < 0.001, and ns indicates no statistical significance.

The viability of *N*. *caninum* was evaluated over a 4 h period using Hoechst 33342 and SYTOX orange dyes. ATA significantly decreased the number of viable *N*. *caninum* at 4 h, with an evident concentration-dependent effect ( Figure 4A–L). Furthermore, statistical analysis indicated that the antiparasitic effect of ATA was time- and dose-dependent within the first 4 h ( Figure 4M).

### ATA disrupts the ultrastructure of *N*. *caninum*

**Figure 5:**
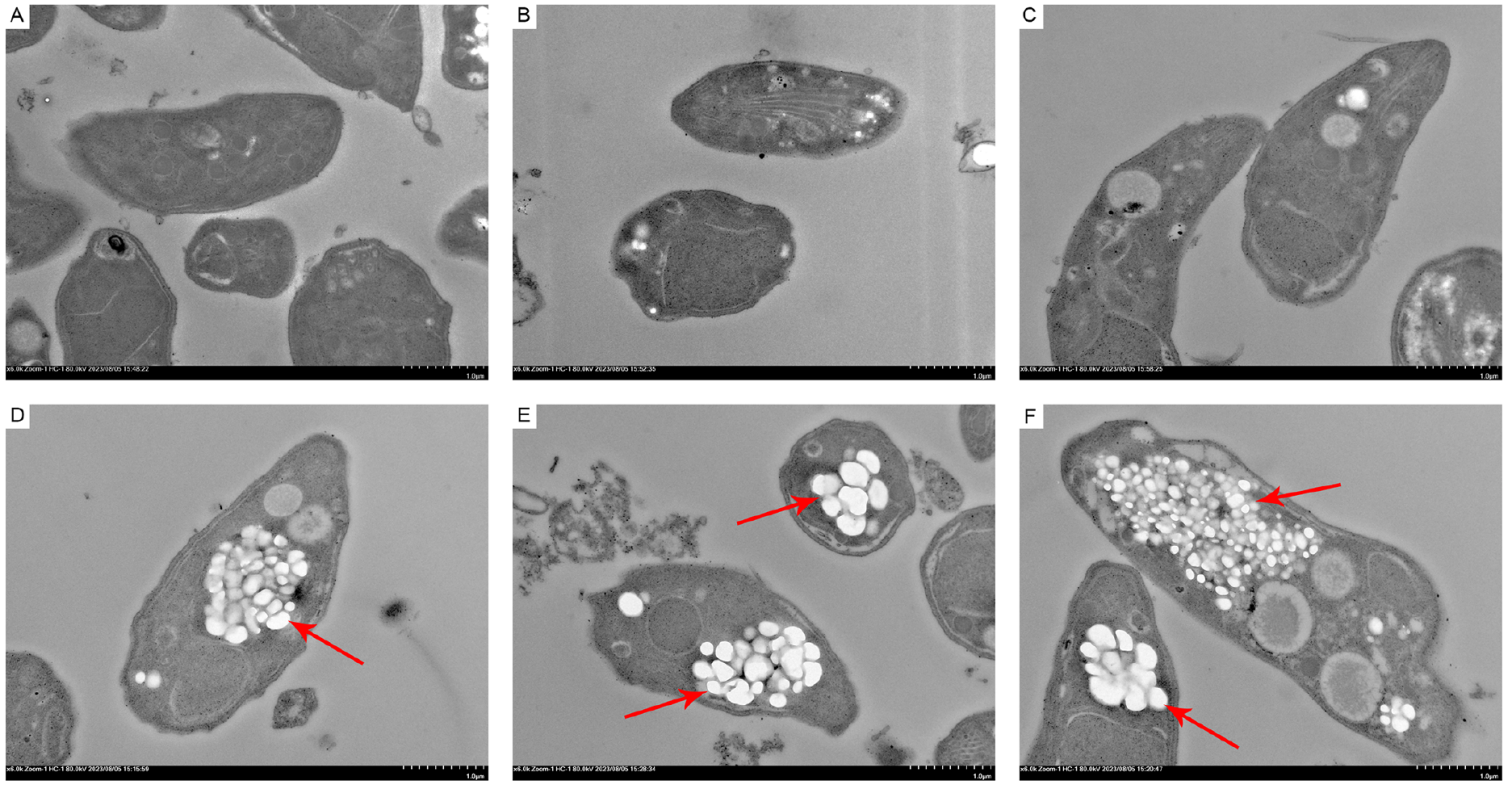
Figure 5 ATA disrupts the ultrastructure of *N*. *caninum* (A–C) Normal ultrastructure of N. caninum tachyzoites. N. caninum is a crescent, oval, or round shape with intact internal organelles and dense cytoplasm. (D–F) Ultrastructure of N. caninum tachyzoites treated with 40 μM ATA for 4 h. ATA disrupts the normal morphology of N. caninum, causing parasite swelling and the formation of numerous vacuoles. Additionally, the loss of conoids and rhoptries as well as the disintegration of parasites were observed. Lesions are indicated by red arrows.

TEM was employed to examine the ultrastructures of *N*. *caninum* tachyzoites. Typically, these tachyzoites exhibited a crescent shape with intact organelles, indicative of normal morphology and internal structure ( Figure 5A–C). However, upon ATA exposure, the cell membranes appeared disrupted or indistinct, with large vacuoles and a loss of cytoplasmic content, and some nuclei exhibited abnormal shapes ( Figure 5D–F).

### ATA enhances body weight in *N*. *caninum*-infected mice

**Figure 6:**
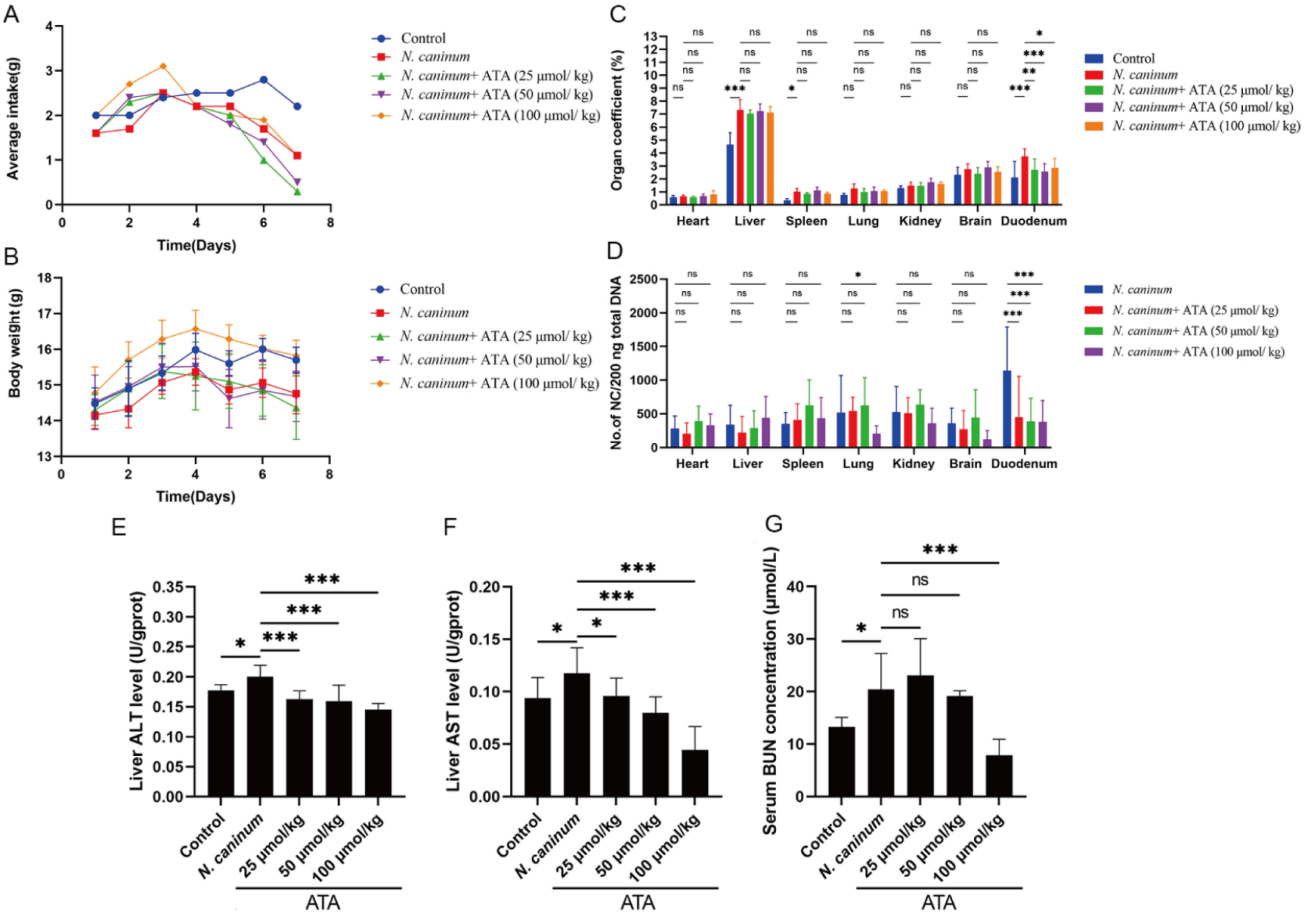
Figure 6 Effects of ATA on *N*. *caninum*-infected mice (A,B) Effects of ATA on the average feed intake and body weight of N. caninum-infected mice. The mice were intraperitoneally inoculated with 1 × 105 tachyzoites of N. caninum, which were then suspended in 100 μL of normal saline solution. The control cohort received an equivalent volume of saline solution. Following infection, the treatment group was administered with oral doses of ATA (25, 50, and 100 μmol/kg) in 100 μL volumes daily for seven consecutive days. (A) Average feed intake of the mice. (B) Body weights of the mice. (C,D). ATA diminishes the organ coefficient and N. caninum load in the lungs and duodenum. (C) Organ coefficient of mouse tissues. (D) N. caninum loads within mouse tissues. (E,F). ATA protects liver and kidney function in N. caninum-infected mice. (E) ALT levels in the liver. (F) AST levels in the liver. (G) Serum BUN levels. Data are presented as the mean ± SD (n = 5). *P < 0.05, ***P < 0.001, and ns indicates no statistical significance.

To further substantiate the efficacy of ATA against *N*. *caninum* infection, the impact of ATA on the body weight of infected mice was evaluated. Compared with the control group, *N*. *caninum* infection resulted in a decrease in the average feed intake and body weight of the mice. Administration of ATA, particularly at a dosage of 100 μmol/kg, substantially improved the appetite of the mice during the early stages of infection within the initial three days, with the body weight of the ATA-treated mice surpassing that of the *N*. *caninum*-infected mice. By the end of the study, the body weight in the 100 μmol/kg dose group was comparable to that in the control group and significantly greater than that in the infected group ( Figure 6A,B).

### ATA diminishes the organ coefficient and parasite loads in the lungs and duodenum

*N*. *caninum* infection significantly increased the organ coefficients of the liver, spleen, and duodenum. In contrast, ATA treatment markedly reduced the organ coefficient of the duodenum ( Figure 6C). Notably, ATA treatment significantly reduced the parasite loads in the duodenum. Additionally, a dosage of 100 μmol/kg led to a significant decrease in the parasite loads within the lungs ( Figure 6D), highlighting the potential of ATA in mitigating the pathological effects of *N*. *caninum* infection in mice.

### ATA protects liver and kidney function in *N*. *caninum*-infected mice

ALT and AST serve as indicators of liver injury, whereas BUN is indicative of kidney function. Infection with *N*. *caninum* significantly elevated the levels of ALT and AST in the liver and the serum level of BUN. However, ATA significantly decreased the levels of ALT, AST, and BUN ( Figure 6E–G), suggesting that it preserves liver and kidney function in *N*. *caninum*-infected mice.

### ATA ameliorates pathological effects induced by *N*. *caninum* infection in mice

**Figure 7:**
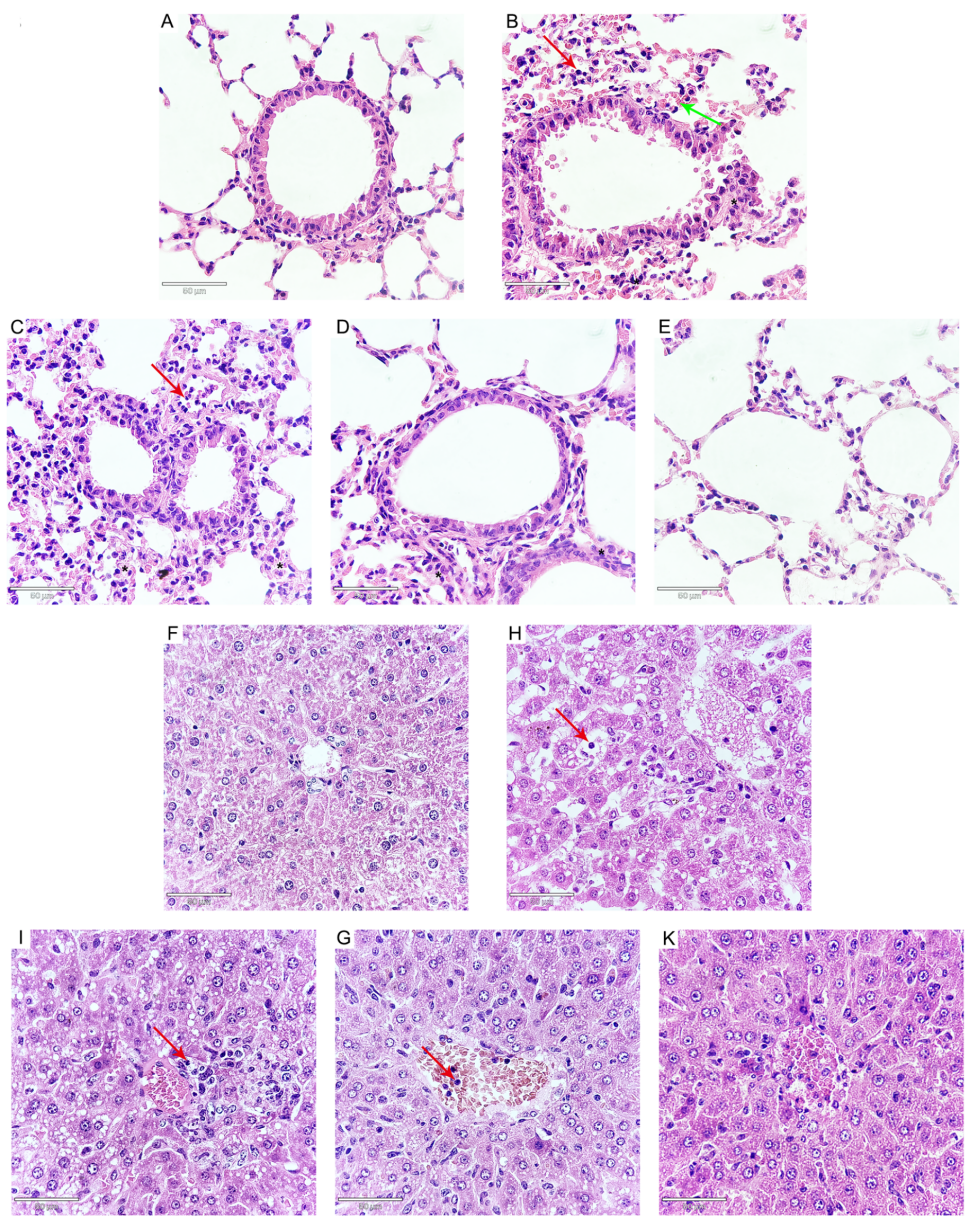
Figure 7 Effects of ATA on *N*. *caninum*-induced pathological changes of lungs and livers (A–E) ATA ameliorates N. caninum-induced pathological changes in the lungs of mice (400×). (A) Control. (B) N. caninum. (C) N. caninum + ATA (25 μmol/kg). (D) N. caninum + ATA (50 μmol/kg). (E) N. caninum + ATA (100 μmol/kg). (F–K) ATA ameliorates N. caninum-induced pathological changes in the liver (400×). (F) Control groups. (H) N. caninum. (I) N. caninum + ATA (25 μmol/kg). (G) N. caninum + ATA (50 μmol/kg). (K) N. caninum + ATA (100 μmol/kg). The red arrow indicates inflammatory cell infiltration. The green arrows indicate hyperemia. Asterisk indicates pulmonary interstitial edema.

**Figure 8:**
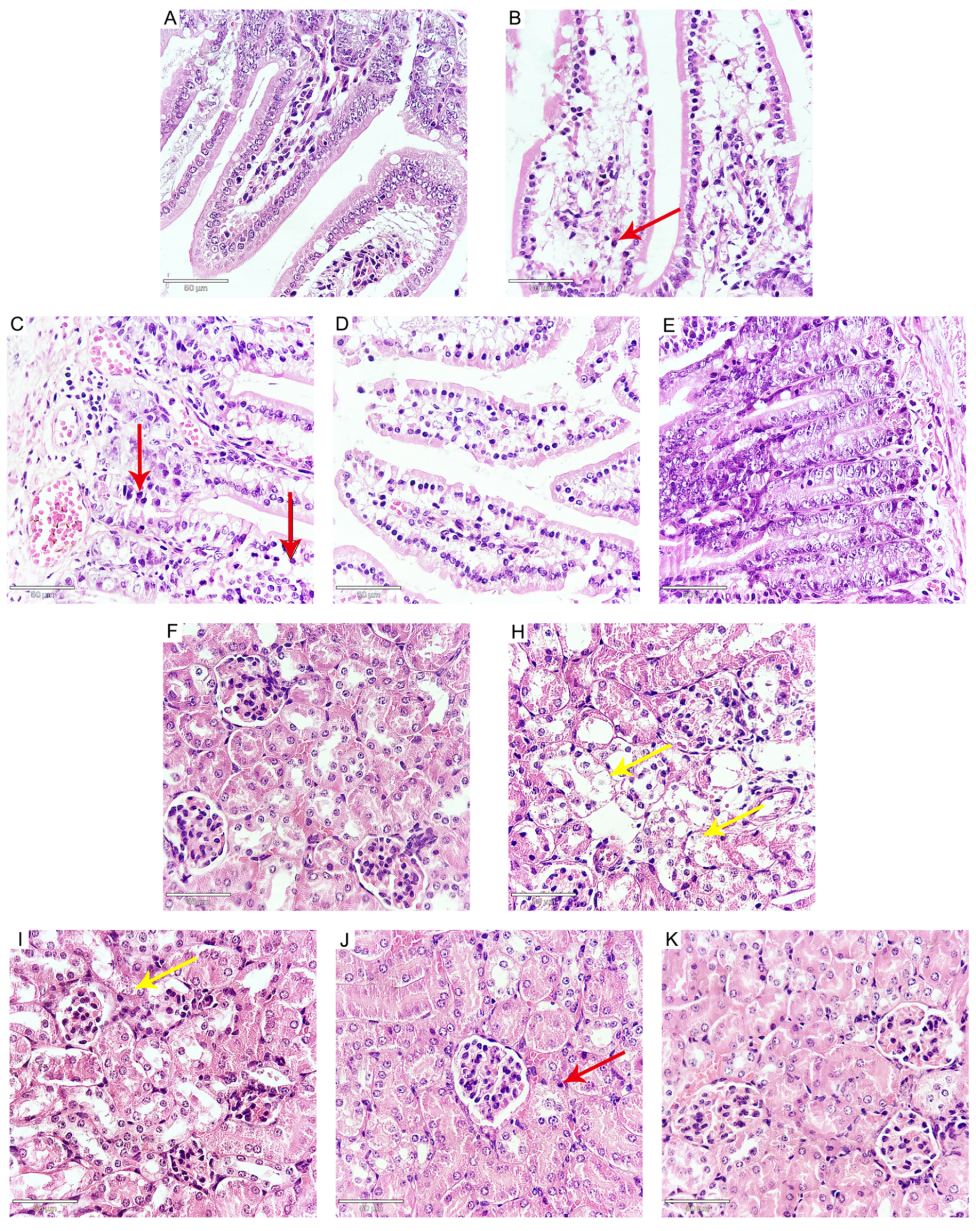
Figure 8 Effects of ATA on *N*. *caninum*-induced pathological changes of duodenum and kidney (A–E) ATA ameliorates N. caninum-induced pathological changes in the duodenum in mice (400×). (A) Control. (B) N. caninum. (C) N. caninum + ATA (25 μmol/kg). (D) N. caninum + ATA (50 μmol/kg). (E) N. caninum + ATA (100 μmol/kg). (F–K) ATA ameliorates N. caninum-induced pathological changes in the kidney (400×). (F) Control. (H) N. caninum. (I) N. caninum + ATA (25 μmol/kg). (J) N. caninum + ATA (50 μmol/kg). (K) N. caninum + ATA (100 μmol/kg). The red arrow indicates inflammatory cell infiltration. Yellow arrows show the shedding of epithelial cells and cellular vacuolization.

Histopathological examination revealed that *N*. *caninum* infection resulted in a variety of pathological changes compared with those in the control group. The observed changes included interstitial edema, an enlargement of interstitial spaces, and hyperemia, accompanied by the infiltration of inflammatory cells. Notably, alveolar cavities in the lungs exhibited signs of collapse, and in some cases, there was a complete loss of the alveolar septum in the lungs ( Figure 7A–E). The liver exhibited edema, inflammatory cell infiltration, and disarray of the hepatic cords ( Figure 7F–K). In the duodenum, there was intestinal villus edema, epithelial thinning, and inflammatory cell infiltration ( Figure 8A–E). Furthermore, the kidneys demonstrated edema and a disarray of renal tubules, along with the shedding of epithelial cells and cellular vacuolization ( Figure 8F–K). Notably, treatment with ATA significantly attenuated these *N*. *caninum*-induced pathological changes (Figures 7 and 8). Collectively, these results indicate that ATA has the potential to protect mice from the detrimental effects of *N*. *caninum* infection.

## Discussion

*N*. *caninum* is an intracellular parasitic protozoon that infects the nucleated cells of mammals. It primarily causes *neosporosis*, which leads to reproductive obstacles such as abortion during pregnancy in dairy cows and beef cattle, resulting in significant economic losses in the cattle industry [ 1, 4]. Additionally, *N*. *caninum* also infects sheep and may be a major cause of abortion in sheep [ 31, 32]. Vaccines are effective means of preventing infection; however, there is currently a lack of commercially available anti- *Neosporosis* vaccines [ 33, 34]. Although there are several commercial chemical drugs for treating *Neosporosis* at present, no specific drug has been developed yet. Therefore, the development of novel anti- *N*. *caninum* drugs is crucial.

ATA is a polyaromatic carboxylic acid derivative with various biological activities. It acts as a DNase inhibitor, reducing the survival rate of African trypanosomes by impeding their immune escape mechanism [16]. Furthermore, ATA has been demonstrated to have excellent effects against *Cryptosporidium parvum* ( *C*. *parvum*), directly killing its sporozoites and oocysts, and has shown excellent anti- *C parvum* effects in C57BL/6 mice [17]. ATA may also target other proteins or cells related to *N*. *caninum* activity; however, owing to its diverse biological activity, the mechanism underlying its anti- *N*. *caninum* effect remains to be further investigated.

In this study, we initially evaluated the influence of different concentrations of ATA on the viability of bovine macrophages. The viability of the cells was significantly reduced by 80 μM ATA, while the survival rate remained at approximately 90%. We subsequently investigated the effects of different ATA concentrations (20, 40, and 80 μM) on *N*. *caninum*. qPCR analysis was employed to investigate the effect of ATA on the parasite load. Notably, 80 μM ATA effectively inhibited *N*. *caninum* proliferation; however, it also led to dysregulation of cytokine expression in macrophages. Conversely, 40 μM ATA had no significant influence on macrophages but effectively inhibited *N*. *caninum* proliferation in macrophages. ATA also inhibited *N*. *caninum*-induced upregulation of cytokines at the mRNA level in bovine macrophages. Moreover, CLSM imaging revealed that ATA not only reduced the overall quantity but also diminished the individual numbers of *N*. *caninum* within the macrophages. In addition to its impact on intracellular parasites, ATA also reduced the survival rate of extracellular *N*. *caninum* by approximately 20% within a span of 4 h, suggesting its potential as an inhibitor even outside the cellular environment. The activity of ATA on *N*. *caninum* is consistent with previous research conducted on *C*. *parvum* [17]. Furthermore, TEM examination confirmed that ATA effectively disrupted the structural integrity of *N*. *caninum* tachyzoites, reaffirming the above experimental results. To further validate the efficacy of ATA against *N*. *caninum* infection, we evaluated its impact on body weight, food intake, parasite loads, biochemical parameters, and pathology *in vivo*. We observed a downwards trend in the AST, ALT, and BUN levels in the ATA treatment group compared with those in the control group; importantly, there was no statistically significant difference between the two groups. These results confirmed the successful establishment of a murine model of *N*. *caninum* infection, and ATA significantly improved body weight, reduced parasite loads in the lungs and duodenum, and mitigated the pathological effects induced by *N*. *caninum* infection.

In conclusion, the findings presented here provide compelling evidence supporting the potential of ATA as a drug of bovine neosporosis and highlight its promising role as a valuable addition to current anti- *N*. *caninum* agents. Nonetheless, additional research should be conducted to elucidate the anti- *N*. *caninum* activity of ATA in cattle.
